# Mutation of *gabra1* is associated with hypermotility and abnormal expression of proteins critical for ion homeostasis and synaptic vesicle transport

**DOI:** 10.1101/2023.01.27.525860

**Published:** 2023-01-27

**Authors:** Nayeli G. Reyes-Nava, Isaiah Perez, Brian Grajeda, Igor L. Estevao, Cameron C. Ellis, Sourav Roy, Anita M. Quintana

**Affiliations:** 1 Department of Biological Sciences, Border Biomedical Research Center, The University of Texas at El Paso, El Paso, Texas, United States of America

## Abstract

Mutation of the *GABRA1* gene is associated with neurodevelopmental defects and epilepsy phenotypes. *GABRA1* encodes for the α1 subunit of the gamma-aminobutyric acid type A receptor (GABA_A_R), which regulates the fast inhibitory impulses of the nervous system. Multiple model systems have previously been developed to understand the mechanism by which mutations in *GABRA1* cause disease, but these models have produced complex and incongruent data. Thus, additional model systems are required to validate and substantiate previously published results. We investigated the behavioral patterns associated with a non-sense mutation of the zebrafish *gabra1* (*sa43718* allele) gene. The *sa43718* allele has a 90% decrease in total *gabra1* mRNA expression, which is associated with light induced seizure-like behavior. Mutation of *gabra1* was accompanied by increased mRNA expression of *gabra4,* which encodes for the alpha-4 subunit of the GABA_A_R. Despite increased expression at the RNA level, Gabra4 protein was not increased according to proteomics analysis. Thus, implying that RNA expression patterns of alpha sub-units may not accurately reflect the mechanism underlying seizure. Interestingly, proteomics analysis identified significant enrichment of genes that regulate proton transport, ion homeostasis, vesicle transport, and mitochondrial protein complexes. Collectively, our analysis validates that mutation of *gabra1* results in seizure like phenotypes and provides a blueprint of putative proteins which may mediate these phenotypes *in vivo*.

## Introduction

The gamma-aminobutyric acid type A receptor (GABA_A_R) is a multi-subunit ion channel that mediates inhibitory synapses of the nervous system. The GABA_A_R can be composed of unique combinations of any of the following: 6 α subunits, 3 β subunits, 3 γ subunits, and one ε, δ, θ, or π subunits. The most common GABA_A_R in mammals consists of 2 α subunits, 2 β subunits, and γ subunit. Each of the subunits is encoded by an independent gene, which are located on different chromosomes. Mutations in many of these subunits have been associated with seizure phenotypes in humans [1] and most recently we used whole exome sequencing to identify a putative heterozygous mutation in the *GABRA1* gene (c.875C>T) associated with seizure phenotypes [2]. *GABRA1* encodes for the α1 subunit of the GABA_A_R and to date, over 30 different variants in the *GABRA1* gene have been reported and associated with neurological disorders and neurodevelopmental defects [2]–[11].

Because of its indicated role in neural development, several murine models have been developed to investigate the physiological and behavioral functions of *GABRA1* in mammals, however, the phenotypic outcomes are heterogeneous. For instance, deletion of *Gabra1* or the knock-in of the p.Ala332Asp allele cause behavioral phenotypes that range from tremors/absence-like seizures to the development of myoclonic seizures at postnatal stages [12]–[15]. Noteworthy, these phenotypes are generally strain, age, and sex specific, which makes interpretation of results difficult. Additional zebrafish models have been characterized including a germline mutant and a morpholino mediated transient knockdown. These two studies yielded contrasting results whereby hypermotility was observed in a nonsense mutant of *gabra1* and hypomotility was observed after transient knockdown [2], [16]. Thus, additional complementary models are warranted.

In 2016, the zebrafish mutation project was completed by the Wellcome Sanger Institute, where over 40,000 mutant alleles, covering 60% of zebrafish protein-coding genes, were generated [17]. However, only a small number of alleles have been characterized or associated with a specific phenotype. An allele carrying a nonsense mutation in the *gabra1* gene (sa43718) was generated and remains uncharacterized. We hypothesized that a more comprehensive analysis of this allele coud discern the disparate phenotypes observed across other zebrafish approaches. We therefore performed behavioral and molecular analysis of the sa43718 allele.

Our results indicate that homozygous *gabra1*^*sa43718/sa43718*^ larvae show hyperactive locomotion (seizure-like behavior) upon a light stimulus. These behavioral changes are associated with a moderate increase in the expression of *gabra4* transcript, but no change at the level of Gabra4 protein expression. However, differential expression of proteins identified via proteomics approaches revealed abnormal expression of proteins that regulate proton transport, sodium ion homeostasis across the plasma membrane, and the mitochondrial inner membrane protein complex and respiratory chain.

## Methods

### Experimental model and animal husbandry

The *gabra1*^*sa43718*/+^ allele (N=10 female fish) was obtained from Sanger Institute through the Zebrafish International Resource Center (ZIRC). Female carriers were outcrossed with wildtype (AB) males to generate independent families of heterozygous male and female carriers. For all experiments, zebrafish larvae were obtained by mating adult heterozygous *gabra1*^*sa43718/+*^ fish. Collected zebrafish embryos were maintained in E3 media (5mM NaCl, 0.17mM KCl, 0.33mM CaCl, 0.33mM MgSO4, 0.05% methylene blue, pH 7.4) at 28°C under 14:10 light: dark cycle. All experimental procedures were performed at 5 days post fertilization (DPF). All animals were maintained and used in accordance with the guidelines from the University of Texas at El Paso Institutional Animal Care and Use Committee (Animal Protocol Number 811869–5). Euthanasia and anesthesia were performed according with the American Veterinary Medical Association (AVMA) guidelines for the euthanasia, 2020 edition.

### Genotyping

Genotyping of the *gabra1*^*sa4371/sa4371*^ allele was performed by PCR amplification and restriction enzyme digest. Adult fin clips and larval tails were lysed in lysis buffer (30ul) (50mM NaOH) for 5 minutes at 95°C followed by 10 minutes at 4°C. DNA was then neutralized by addition of 500mM Tris-HCL solution (6ul) and used directly for PCR. The fragment of interest (237bp) was amplified by standard PCR at an annealing temperature of 60°C (forward: TTGTGACTCAAAGCCACGAG and reverse: TGAGACGAGAACCATCGTCA). PCR amplicon containing the *sa43718* allele region, was digested with XhoI (NebBiolabs) at 37°C according to manufacturer’s guidelines. The mutation present in the *sa43718* inhibits cleavage by XhoI allowing differentiation of wildtype, heterozygous, and homozygous offspring.

### Behavioral analysis and pentylenetetrazole (PTZ) treatment

Behavioral analysis was performed using the ZebraBox (ViewPoint Behavioral Technology, Montreal, Canada). Briefly, embryos were obtained from natural spawning and raised to 5 DPF. Larvae (5 DPF) were individually tracked for swim speed and total distance swam in a 96-well plate. The behavioral analysis consisted of 15 minutes divided into 5-minute intervals of dark/light/dark conditions. All larvae were acclimated to the chamber for 1 hour prior to data acquisition [2]. Data was collected each minute, for a total of 15 minutes and total distance traveled (mm) and speed (mm/s) was calculated as previously described [2]. Experiments were performed in biological duplicates using a minimum of N=30 larvae per trial. Treatment with 10mM pentylenetetrazole (PTZ) (Millipore-Sigma) was performed as previously described [2]. Genotyping of each larvae was performed after behavioral analysis.

### Quantitative real time PCR (QPCR)

Total RNA was isolated from brain homogenates at 5 DPF from wildtype, heterozygous, and homozygous larvae with TRIzol reagent (Invitrogen) according to manufacturer’s instructions. RNA was reverse transcribed using the Verso cDNA Synthesis Kit (ThermoFisher Scientific) and total RNA (500ng) was normalized across samples. Gene expression was measured in biological quadruplicates using a pool of at least N=5 larvae per biological replicate. For each individual biological replicate, technical replicates were performed as internal controls. The Applied Biosystems StepOne Plus machine with Applied Biosystems associated software was used for quantitative PCR (qPCR) analysis. Sybr green (ThermoFisher Scientific) based primer pairs were designed for each gene analyzed: *gabra1* (FWD: TCGGGAGTCCAGATTTTGCT, REV: AGAGCGTGTAACCGAAGTCA), g*abra2a* (FWD: GATGGCTACGACAACAGGCT, REV: TGTCCATCGCTGTCGGAAAA), *gabra3* (FWD: GCTGAAGTTCGGGAGCTATG, REV: GGAGCTGATGGTCTCTTTGC), *gabra4* (FWD: GACTGCGATGTACCCCACTT, REV: ATCCAGGTCGGAGTCTGTTG), *gabra5* FWD: CATGACAACACCCAACAAGC, REV: CAGGGCCTTTTGTCCATTTA), *gabra6a* (FWD: TCGCGTACCCATCTTTCTTC, REV: CCCTGAGCTTTTCCAGAGTG), *gabra6b* (FWD: CGGAGGAGTGCTGAAGAAAC, REV: GGGAAAAGGATGCGTGAGTA), *gabrb2* (FWD: CCCGACACCTATTTCCTCAA, REV: TCTCGATCTCCAGTGTGCAG), *gabrg2* (FWD: ACACCCAATAGGATGCTTCG, REV: AGCTGCGCTTCCACTTGTAT). The *rpl13a* gene was used as a reference gene for 2^ΔΔct^ quantification. Statistical analysis performed using a *t-test*.

### Proteomic analysis

#### Protein isolation

For protein analysis, total protein was obtained from a pool of whole brain homogenates (n=9) per genotype. Two biological replicates per genotype were collected. Brains were excised and stored for protein isolation in 1X Cell Lysis Buffer (ThermoFisher Scientific) with protease inhibitors cocktail (ThermoFisher Scientific). DNA was isolated from the tail tissue for genotyping and protein was isolated from brain homogenates using manual homogenization. Protein quantification was performed using Precision Red Protein Assay Reagent (Cytoskeleton) according to manufacturer’s instructions. Samples were then sent to the Biomolecule Analysis and Omics Unit (BAOU) at The University of Texas El Paso for sample processing and proteomic analysis.

#### Sample preparation for proteomics

Proteins were removed from 1X Cell Lysis Buffer by trichloroacetic acid (TCA) protein precipitation. Fifty microliters of 100% TCA (Sigma/Millipore - cat# T6399-5G) was added to 200 μL of sample in 1x Cell Lysis Buffer (Safe Seal microcentrifuge tubes, Sorenson BioScience, cat. No. 12030) and incubated at 4°C for 10 minutes. Total protein ranged from 2 – 5 μg per sample. The precipitated proteins were pelleted by centrifugation for five minutes at 14,000 rcf and the supernatant was discarded. The protein pellet was subsequently washed a total of three times with 200 μL LCMS grade acetone by resuspension and pelleting, the acetone supernatant was discarded each time. Residual acetone was evaporated with a heating block at 95°C for 2 minutes. Samples were stored as a pellet at −80°C until processing for proteomic analysis. Stored pellets were resuspended in 8M Urea prior and subjected to enzymatic tryptic digestion using the PreOmics iST sample preparation kit (catalog no. iST 96x P.O.00027). Briefly, denaturation, alkylation, and reduction were performed with 10 μL LYSE buffer per 1 μg of protein and heated at 80 °C for 20 min while being mixed every 5-minutes. Remaining droplets on the cap were given a brief centrifugation (RT; 300 rcf; 10 sec) and samples were sonicated ten times at 30-second on/off intervals for a total of 5 minutes. Fifty microliters of resuspended DIGEST solution were added to the samples for protein digestion. Sample-filled microtubes were gently vortexed, centrifuged and kept for 90 minutes at 37°C in a heating block. Throughout the 90-minute incubation period, samples were gently mixed every 10 minutes. One hundred microliters of STOP solution were added and mixed by pipetting ten times. Samples were then transferred to the cartridge and centrifuged for 1 minute at 3,800 rcf, followed by subsequent 200 μL washes with WASH 1 and WASH 2 solutions according to the manufacturer’s instructions. Peptides were eluted in two cycles of 100 μL of ELUTE solution and dried completely in a vacuum evaporator (Savant; Thermo Fisher Scientific) for 90 minutes at 45 °C and 100-mTorr and stored at −80°C until LC-MS/MS acquisition.

#### Liquid chromatography–tandem mass spectrometry (LC-MS/MS)

Peptides were resuspended in 4% acetonitrile (ACN) with 0.1% formic acid (FA) at a concentration of 1μg/μL. Peptides were separated by a Dionex Ultimate 3000 UHPLC system (Thermo Scientific) tandem with a Q-Exactive Plus Hybrid Quadrupole-Orbitrap Mass Spectrometer (Thermo Scientific) with Xcalibur software (v. 3.0.63) for data acquisition in positive mode. Peptides were separated on a C18 Acclaim PepMap nanoLC column (75 μm × 50 cm nanoViper, PN 164942, Thermo Fisher Scientific) equilibrated with 4% solvent B (99.9% acetonitrile, 0.1% formic acid) and 96% solvent A (99.9% H2O, 0.1% formic acid) kept at 55°C throughout the entire acquisition. One microliter of peptides was loaded onto the column for 15-minutes at a flow rate of 0.5 μL/min and eluted with a multi-step gradient. The flow rate was reduced to 0.3μL/min over the course of 15 min, and solvent B was set to 20% over 100-minutes, then increased to 32% and maintained for 20-minutes before increasing to 95% over 1-minute. The column was washed with 95% solvent B at a flow rate of 0.4 μL/min for 4-minutes to remove/clean any remaining peptides. The column was then re-equilibrated with 4% solvent B at 0.5μL/min until a 180-minutes total runtime. Blank injections were added after each biological replicate, using a 60-minute two sawtooth gradient from 4–95% solvent B, and column re-equilibration at 4% solvent B. The mass spectrometer was set to top10 data-dependent acquisition (DDA), with a scan range of 375 to 1500 m/z and a full MS resolution of 70,000; AGC target 3e6. Ions were fragmented with NCE at 27 and collected with an AGC target of 1e5 at 17,500 resolution, 2 m/z isolation window, maximum IT of 60 ms. Charged exclusion ions were unassigned, 1, 6–8, and >8 charges.

### Bioinformatics data analysis

After proteomics analysis, Proteome Discover (PD) 2.5.0.400 (Fisher Scientific) was utilized to identify the proteins from each peptide mixture. The database for Danio rerio was downloaded from UniProtKB; http://www.uniprot.org/ on 21 October 2021 with a database 61,623 sequences. A contaminant dataset was run in parallel composed of trypsin autolysis fragments, keratins, standards found in CRAPome repository and in-house contaminants. PD analysis parameters are as follows: false-discovery rate (FDR) of 1%, HCD MS/MS, fully tryptic peptides only, up to 2 missed cleavages, parent-ion mass of 10 ppm (monoisotopic); fragment mass tolerance of 0.6 Da (in Sequest) and 0.02 Da (in PD 2.1.1.21) (monoisotopic). Two-high confidence peptides per protein were applied for identifications. PD dataset was processed through Scaffold Q+S 5.0.1. Scaffold (Proteome Software, Inc., Portland, OR 97219, USA) was used to probabilistically validate protein identifications derived from MS/MS sequencing results using the X!Tandem and Protein Prophet. Data was transferred to Scaffold LFQ (Proteome Software, Portland, Oregon, USA) which was used to validate and statistically compare protein identifications derived from MS/MS search results. A protein threshold of 95%, peptide threshold of 95%, and a minimum number of 2 peptides were used for protein validation. Normalized weighted spectral counts were used when comparing the samples. To ascertain p-values, Fisher’s Exact was run with a control FDR level q * .05 with standard Benjamini-Hochberg correction.

## Results

### Characterization of the sa43718 allele

**Fig 1.**
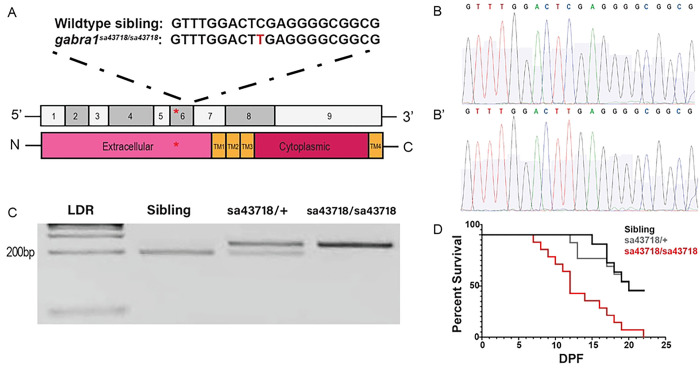
The sa43718 allele carries a single non-sense mutation in the *gabra1* gene. (A) Schematic representation of the *gabra* ^*sa43718*^ allele. Homozygous individuals of the sa43718 allele (*gabra1*^*sa43718/sa43718*^) carry a single base pair substitution (C>T) in the *gabra1* gene (ENSDART0000010000), predicted to result in a premature stop. Schematic of exon structure of the *gabra1* gene, not to scale. The sa43718 site is indicated with a red asterisk within exon 6, which encodes the extracellular domain of Gabra1. (B-B’) Chromatograph showing confirmation of the sa43718 allele through Sanger sequencing of *gabra1*^*sa43718/+*^ offspring. (B) Wildtype sibling sequence. (B’): *gabra1*^*sa43718/sa43718*^ sequence. (C) Restriction enzyme digest gel showing digestion of PCR fragments of genomic *gabra1.* The sa43718 allele abolishes the Xhol recognition site. Therefore, digestion of homozygous (sa43718/sa43718) sample results in an uncut fragment, whereas digestion of wildtype (sibling) sample results in a fully digested fragment. LDR: 1kb plus ladder. (D) The offspring of *gabra1*^*sa43718/+*^ fish was genotyped by tail-clipping at 3 days post fertilization (DPF). Larvae were raised separately according to their genotypes (sibling n=11, sa43718/+ n=13, sa43718/sa43718 n=14), and their survival was monitored until day 22. Premature death of homozygous carriers of the sa43718 allele (sa43718/sa43718*)* was observed starting at day 7 post fertilization, when compared to the wildtype (sibling) and heterozygous (sa43718*/+)* siblings.

The sa43718 allele results in a single base pair substitution in exon 6, which is predicted to result in a premature stop codon (Fig 1A). Exon 6 encodes part of the extracellular domain. We used Sanger sequencing to validate the single base pair substitution shown in Fig 1B (Sibling) &B’(sa43718). Based on the sequence change present in the sa43718 allele, we developed a restriction enzyme based genotyping strategy. The sa43718 allele abolishes an XhoI cleavage site and therefore cannot be cleaved by XhoI in a restriction digest. We performed PCR amplification using primers that flank the substituted nucleotide and used restriction digest to identify wildtype, heterozygous, and homozygous larvae. DNA from wildtype siblings was completely digested as predicted (Fig 1C, lane 2), while DNA from homozygous larvae was completely undigested, and DNA from heterozygous carriers was partially digested (Fig 1C, lane 4 and 3, respectively).

Homozygous mutation in zebrafish *gabra1* has been associated with early lethality. Consequently, we performed a survival assay and monitored the survival for 22 DPF. We observed a 50% survival rate in wildtype and heterozygous siblings, but all homozygous carriers of the sa43718 allele died by 22 DPF (Fig 1D). The remaining heterozygous individuals survived into adulthood.

### Expression of GABA_A_ receptor subunits in the sa43718 allele.

**Fig 2.**
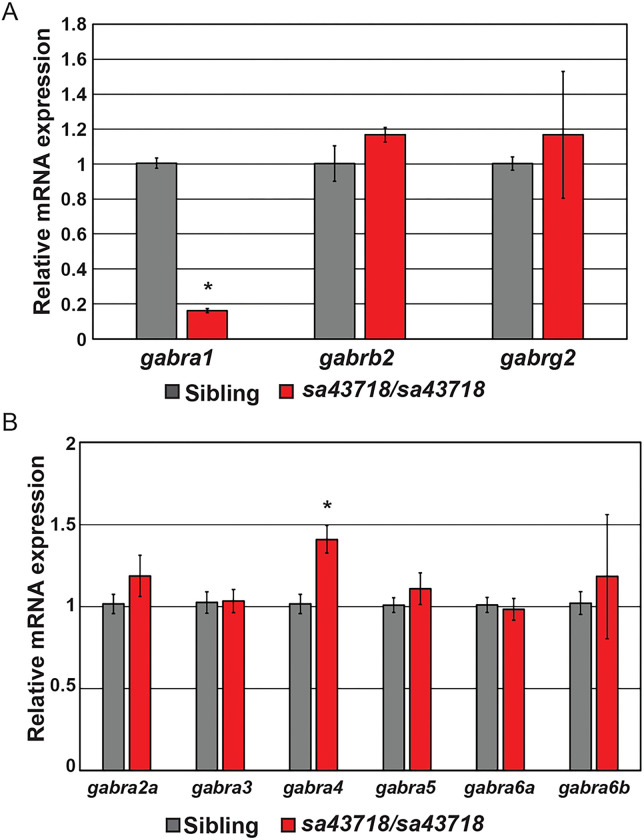
Non-sense mutation of *gabra1* results in decreased expression of *gabra1*. (A) Quantitative real time PCR (qPCR) analyzing the relative mRNA expression of major GABA_A_R subunits, α1 (*gabra1),* β2 *(gabrb2)* and, γ2 (*gabrg2)* at 5 days post fertilization (DPF). n≥5 larvae per group with five biological replicates. Error bars represent the standard error of the mean of biological replicates. *p=1.00511E-07. (B) Relative expression of genes encoding for alpha type subunits, *gabra2a* (α2), *gabra3* (α3), *gabra4* (α4), *gabra5* (α5), *gabra6a* (α6a), and *gabra6b* (α6b), was analyzed in wildtype siblings (sibling) and homozygous (sa43718/sa43718) mutants. n≥5 larvae per group in four independent biological replicates. Error bars represent the standard error of the mean. *p=0.002884.

Based on our previous studies, we hypothesized that the expression of other GABA_A_R receptor subunits were disrupted in the sa43718 allele. We first measured the expression of β2 and γ2 subunits, as these are present in the most common form of the GABA_A_R receptor, alongside α1. We validated that the sa43718 allele reduced the expression of *gabra1* (Fig 2A), but that decrease was not associated with decreased expression of β2 and γ2 transcripts (Fig 2A). We therefore hypothesized that these subunits were in complex with other α subunits to compensate for the loss of α1. We measured the expression of α2–6 (including 6a and 6b due to gene duplication). We observed a mild increase in *gabra4* (α4) gene expression, which was statistically significant (Fig 2B). However, we did not detect a significant difference in any other α subunit (Fig 2B).

### Mutation of *gabra1* results in seizure-like behavior

**Fig 3.**
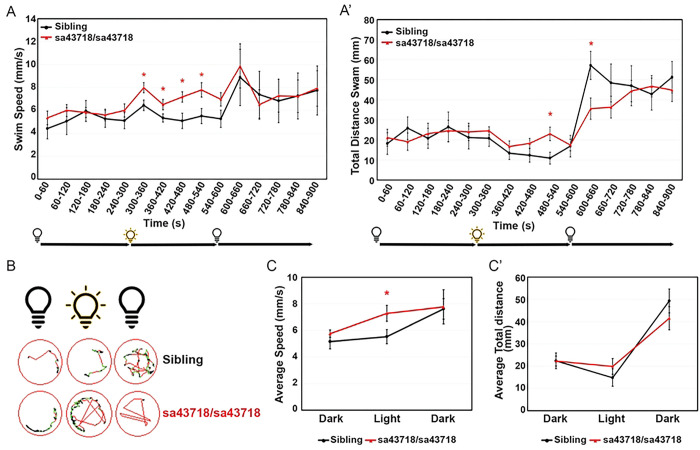
*gabra1*^*sa43718/sa43718*^ larvae undergo seizure-like behavior upon light stimuli. (A & A’) Behavioral analysis of sa43718 allele and wildtype siblings at 5 days post fertilization. (A) Swim speed (mm/s) over 15 minutes (900 seconds) in dark-light-dark transitions [5 minutes (0–300 seconds) in the dark, 5 minutes (300–600 seconds) in the light, and 5 minutes (600–900 seconds) in the dark) was analyzed using the Zebrabox technology. Data was collected every minute (60 seconds). *gabra1*^*sa43718/sa43718*^ larvae undergo hyperlocomotion upon light stimuli when compared to their wildtype siblings (Sibling). *p<0.05. (A’) Total distance swam over 15 minutes in dark-light-dark transitions. Homozygous carriers (sa43718/sa43718) larvae showed increased distance swam across the light phase, showing a statistically significant increase at seconds 480–540 (minute 9), when compared to their wildtype counterparts (sibling). *gabra1*^*sa43718/sa43718*^ larvae then show decreased distance swam under dark conditions (600–660s) when compared to their wildtype siblings (sibling) *p<0.05. (B) Representative images of swimming track of a single larvae, representing each genotype, generated from the Viewpoint Zebralab Tracking software in dark (180–240s), light (480–540s), and dark (600–660s) conditions. Green lines indicate movements between 4–8mm/s and red lines indicate burst movements (>8mm/s). (C) Average total speed representing overall behavioral responses in dark-light-dark conditions of *gabra1* wildtype (sibling) and homozygous (sa43718/sa43718) larvae. Homozygous carriers (sa43718/sa43718) showed increased average swim speed in light conditions when compared to their wildtype (sibling) counterparts. (C’) Average total distance swam representing overall behavioral responses in dark-light-dark conditions of *gabra1* wildtype (sibling) and homozygous (sa43718/sa43718) larvae. No statistically significant changes were observed in homozygous larvae (sa43718/sa43718) when compared to their wildtype siblings for average distance measurements. All behavioral analysis was performed using a minimum of 30 larvae per biological replicate, over three independent biological replicates. Representative analysis of a single biological replicate is shown here. Sibling (n=13) and *gabra1*^*sa43718/sa43718*^ (n=26).

We have previously reported hypoactivity at 5 DPF upon knockdown of *gabra1* [2]. In contrast, seizure-like behavior at juvenile stages has been reported in a zebrafish model of *gabra1* loss of function [16]. To begin to rectify these differences, we monitored swimming behavior after a light stimulus, which has been shown to induce seizure like behavior in a previous allele of *gabra1* mutation. We measured baseline activity for a period of 5 minutes in the dark after a 1-hour acclimation period. During this time, we did not observe any differences in baseline swimming patterns according to total distance swam (mm) or swim speed (mm/s) in the sa43718 allele (Fig 3A&A’). However, after light stimulus, we detected a marked increase in swim speed in the sa43718 allele (Fig 3A&A’). Swim patterns were increased in mutant larvae during the entire light stimulus (Fig 3C&C’). Importantly, increased speed (velocity) was associated with rapid circling behavior, hyperactivity burst, and whirlpool behavior (Fig 3B) indicative of a seizure-like behavior as defined by the zebrafish behavioral glossary [18]. Because we did not observe behavioral changes in heterozygous carriers in comparison to wildtype siblings, all data shown here are from sibling wildtype or homozygous carriers of the sa43718 allele.

### *Gabra1 sa43718* response to PTZ

**Fig 4.**
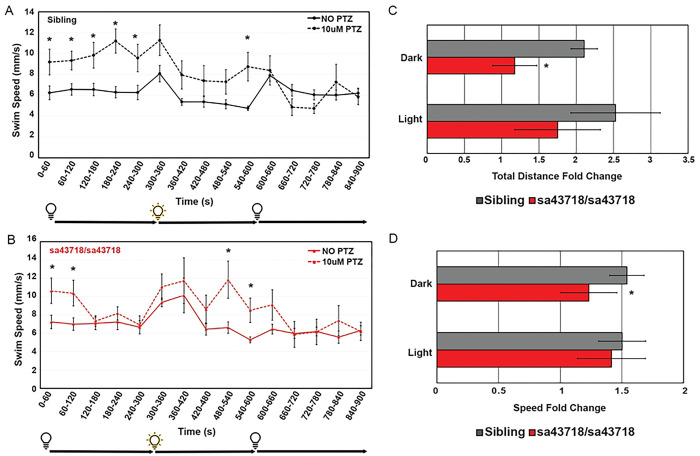
Locomotion response to pentylenetetrazole (PTZ) in the sa43718 allele. (A-B). Behavioral responses of the sa43718 allele at 5 days post fertilization (DPF) were analyzed using the Zebrabox. (A) Comparison of wildtype sibling (Sibling) larvae response to treatment with 10uM PTZ (10uM PTZ) or no treatment (NO PTZ) at 5 days post fertilization. NO PTZ n=18, 10uM PTZ n=16. *p<0.05 (B) Comparison of homozygous carriers (sa43718/sa43718) response to treatment with 10uM PTZ (10uM PTZ) or no treatment (NO PTZ) at 5 days post fertilization. NO PTZ n=13, 10uM PTZ n=14. *p<0.05. Swim speed (mm/s) was analyzed using the distance swam and swimming duration data generated by ViewPoint software. Larvae were monitored for 5 minutes in the dark (0–300 s), 5 minutes in the light (300–600 s), and 5 minutes in the dark (600–900 s). Data was collected every minute (60 s). Error bars represent the standard error of the mean. (C) Average fold change (total distance) response to treatment with PTZ in dark (0–300s) and light (300–600s) conditions by wildtype (sibling) and sa43718/sa43718 mutants. *p=0.002. (D) Average fold change (swim speed) response to treatment with PTZ in dark (0–300s) and light (300–600s) conditions by wildtype (sibling) and sa43718/sa43718 mutants. *p=0.03. Error bars represent standard deviation in C-D.

We have previously demonstrated that reduced *gabra1* expression does not prohibit receptor responses to pentylenetetrazole (PTZ), a GABA_A_R antagonist [2]. We also observed increased expression of *gabra4* and therefore, we sought to understand if the sa43718 allele would respond to PTZ, which may indicate the presence of a functional receptor. As shown in Fig 4A, treatment with PTZ induces increased swimming distance and speed in wildtype larvae. Within the first five minutes post treatment, in dark conditions, wildtype larvae exhibit an average 2-fold change in total distance swam and a 1.54-fold change in swim speed. However, the sa43718 allele had a slightly reduced response to PTZ in the dark accounting for a 1.7-fold change in total distance swam and a 1.2-fold change in swim speed in dark conditions (minutes 1–5) (Fig 4C–D). We continued to monitor the speed and distance swam after light stimulus in PTZ treated larvae. After light stimulus, wildtype sibling larvae showed a 1.5-fold change in average swim speed and a 2.5-fold change in average total distance swam (Fig 4C–D). Whereby, the sa43718 allele exhibited a 1.4-fold change in average swim speed and 1.7-fold change in average distance swam in response to PTZ treatment (Fig 4C–D). The reduced response to PTZ treatment by the sa43718 allele was statistically significant in dark conditions for speed (p=0.03) and distance (p=0.002).

### Proteomic analysis identifies differentially expressed proteins (DEPs) in the sa43718 allele.

**Fig 5.**
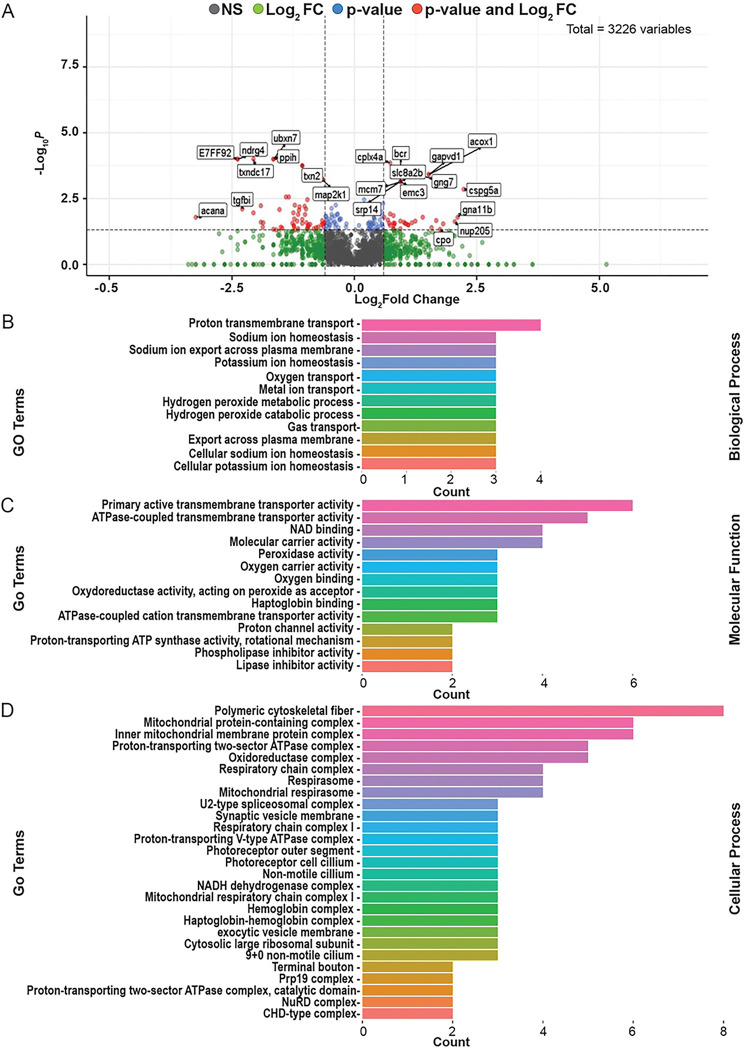
Proteomic analysis of sa43718 allele carriers. (A) Volcano plot of differentially expressed proteins between wildtype siblings and homozygous (sa43718/sa43718) larvae. Volcano plot show −log10 P-values from the normalized proteomics data exported from Scaffold LFQ versus log2foldchange (FC) across each contrast. Thresholds set were p-value ≤ 0.05 and Log2FC ≤ −1.5 or ≥ 1.5. (B-D) Gene Ontology terms enriched by proteins with significant expression (p-value <0.05) between wildtype sibling and sa43718 homozygous mutant larvae at 5 days post fertilization. Terms are separated by (B) Biological Process, (C) Molecular Function, (D) Cellular Process.

To begin to understand the underlying molecular mechanisms by which seizure phenotypes occurred, we performed proteomics analysis. We isolated protein from whole brain homogenates isolated from wildtype and homozygous larvae. A total of 3500 proteins were identified in the wildtype sibling brain homogenate, 3511 were identified in heterozygous animals, and 3552 were identified in the homozygous carriers of the sa43718 allele. A complete list of identified proteins is in S1 file. From these proteins, we identified a total of 173 DEPs in homozygous mutants relative to wildtype clutch mates. Of these 81 were up-regulated and 93 were down-regulated. These DEPs are summarized in the volcano plot shown inf Fig 5A. A complete list of DEPs can be found in file S2. DEPs of interest include Synaptotagmin, Complexin 4a, Dynactin subunit 1, Dynamin GTPase, Secretogranin II, and a Vat1 homolog. Each of these proteins has a function in synaptic fusion, transport, exocytosis, endocytosis, or direct interactions with small synaptic vesicles. Interestingly, we also noted a significant number of DEPs involved in microtubule growth, microtubule binding, vesicle recycling, retrograde transport along microtubules, or microtubule folding. These include microtubule associated protein, RP/EB family member 3b, Dynactin subunit 1, Dynamin GTPase, and Tubulin-specific chaperone A.

We further annotated DEPs by biological process (Fig 5B), molecular function (Fig 5C), and cellular process (Fig 5D). According to biological process, DEPs were clustered in Gene Ontology (GO) groups associated with proton transport including sodium and potassium export across the plasma membrane. Some examples of DEPs in this category include Slc82b, Atp1a1b, Atp1b3a, and Atp1a1a.3. All these DEPs are important for sodium and potassium homeostasis consistent with GO analysis. GO analysis by molecular function also indicated molecular carrier activity and active transport mechanisms, consistent with the GO analysis according to biological process. GO analysis by cellular process indicated DEPs in the respiratory chain, mitochondrial protein containing complexes, the cytoskeleton, proton transport, and exocytosis. These data collectively indicate mitochondrial transport, synaptic vesicle regulation, and proton homeostasis are dysregulated after mutation of *Gabra1*.

## Discussion

The GABA_A_R has previously been associated with seizure and epileptic phenotypes [1], [9], [15]. In support of this notion, we reported a heterozygous missense mutation in humans that is associated with a seizure phenotyes [2]. Multiple model systems have been developed to study early *GABRA1* function. These include mouse and zebrafish models. Survival of homozygous knockouts in mice and zebrafish is compromised [12], [16]. Due to external fertilization and early juvenile survival of homozygous mutants, zebrafish provide a window of opportunity to analyze developmental and juvenile onset of seizures [19]–[24]. However, in previous studies germline nonsense mutation of zebrafish *gabra1* and morpholino mediated knockdown of *gabra1* demonstrated different behavioral phenotypes after a light stimulus [2], [16]. Here we characterized the phenotypes of a novel allele, known as the sa43718 allele. The sa43718 allele is a single base pair change in exon 6, which results in a premature stop codon and leads to a greater than 90% decrease in *gabra1* expression, indicating the possibility of nonsense mediated decay of the mutated transcript. The sa43718 allele reduces viability and homozygous mutants die by 22 DPF. This is consistent with the viability of other zebrafish [16] and mouse models [13].

We observed seizure like (hypermotility, whirlpool behavior) behavior upon light stimuli in the sa43718 allele. These data are supported by previously published works, which established seizure like behavior at the juvenile stage in an additional zebrafish germline mutant [16]. Similar results have also been documented in mouse models of *Gabra1* mutation, although these data are sex and strain specific [12]–[14]. In our experiments, the zebrafish larvae have not yet determined sex and therefore, sex is not a variable considered in our experiments. Hyperactivity and seizure like behavior in the sa43718 allele contrasts with our previous study using morpholino mediated knockdown [2]. There are several reasons for these differences. For example, we validated an approximate 50% knockdown of *gabra1* in morphants, which would most likely reflect a haploinsufficency or dosage effect. In addition, *gabra1* morphants demonstrated with decreased expression of other GABA_A_ receptor subunits. We did not observe statistically significant differences in other subunits, except for the *gabra4* subunit. However, this was only at the mRNA level, as proteomics did not reveal differences in the Gabra4 protein level in the sa43718 allele.

Given the normal expression of other GABA_A_R subunits, it is not surprising that sa43718 larvae responded to PTZ treatment. Mutant larvae were capable of a response to 10uM PTZ, which has been shown to induce seizures in zebrafish [25], [26]. These data are consistent with our previous work, which demonstrated that *gabra1* knockdown did not affect response to PTZ [2]. Additionally, Samarut and colleagues demonstrated that the overall brain morphology of *gabra1* mutant brains was normal. Despite an overwhelming normal morphology, Samarut and colleagues did show 460 differentially expressed genes in *gabra1* mutant brains. Of these genes, 3 GABA_A_R subunits, in addition to *gabra1*, were down regulated [16]. We observed increased expression of *gabra4* here and in a previous study we found *gabra6* to be up regulated at the transcriptional level after morpholino mediated knockdown [2]. These data are partially supported by murine studies that show abnormal expression of receptor subunits. Although data from murine studies are mutant, strain, and gender specific [27], [28]. We performed proteomics analysis to determine if these changes translate to differential expression of proteins. We did not identify a single GABA_A_R subunit differentially expressed in the sa43718 allele at the protein level. These data are consistent with the ability of *gabra1* mutants to respond to PTZ, indicating the presence of a receptor.

Our proteomics analysis identified proteins with functions in synaptic vesicle transport, microtubule organization/growth, mitochondrial function, and regulation of potassium and sodium homeostasis as differentially expressed. A subset of these data are consistent with data from Samarut and colleagues, who noted decreased expression of kinesin motor proteins [16]. However, the functional significance of differential expression of transcripts is unclear because we did not validate up-regulation of Gabra4 protein despite increased transcript. Thus, there are some differences from our protein data relative to previous results. For example, we did not identify statistically significant changes in neuroligins 2 and 4, nor other GABA_A_ subunits. We cannot rule out differential expression of these proteins, however, in our analysis we did not find significant changes in protein expression. Of note, we identified abnormal expression of synaptotagmin, secretogranin II, and synapsin IIb; all of which are important for synaptic vesicle release at the presynaptic membrane. Synapsin I was also downregulated and has been associated with epileptic phenotypes [29]–[32]. Furthermore, we identified downregulation of proteins implicated in mitochondrial complex I deficiency, including NADH:ubiquinone oxidoreductase core subunit S2 and NADH:ubiquinone oxidoreductase core subunit A10. Remarkably, mutations in the genes encoding these proteins are associated with Leigh syndrome, one of the most common mitochondrial diseases, in which epilepsy is a primary phenotype [33], [34]. Interestingly, mutation of *GABRA1* in humans have been prematurely diagnosed as a primary mitochondrial disorder prior to exome sequencing [23]. Interestingly, none of the DEPs discovered inhibited the response to PTZ, a GABA_A_R agonist, suggesting that a residual receptor is present. Nonetheless, our proteomics data implicates neurotransmitter release, vesicle transport, microtubule structure, proton homeostasis, and regulation of mitochondrial proteins as putative underlying mechanisms resulting in seizure-like phenotypes after mutation of *gabra1*.

Here we have characterized an additional germline mutant of the zebrafish *gabra1* gene. We observed early lethality, seizure-like hypermotility after light stimuli, and abnormal expression of proteins involved in vesicle transport, synaptic vesicle release, exocytosis, and endocytosis. Our data support the hypermotility and expression patterns previously described in a secondary zebrafish germline mutant [16]. Collectively, these data suggest that models derived from germline mutation of *gabra1* rather than transient knockdown are more physiologically relevant to human disease.

## Data Availability

All data and reagents are available upon request from the corresponding author.
